# Relationships between experiences of humiliation on social networks, problematic phone use, and aggressive and altruistic behaviors in young adults

**DOI:** 10.3389/fpsyg.2024.1368336

**Published:** 2024-06-17

**Authors:** Clara López-Mora, Gustavo Carlo, Irene Huguet López, Francisco Javier González-Blázquez, Elia Oliver Gasch

**Affiliations:** ^1^Faculty of Health Sciences, Universidad Europea de Valencia, Valencia, Spain; ^2^School of Education, University of California, Irvine, Irvine, CA, United States

**Keywords:** cyber victimization, problematic smartphone use, aggressive behaviors, altruistic prosocial tendencies, mediated effects

## Abstract

The purpose of this study was to analyze the relationships between cybervictimization in social networks, problematic smartphone use, aggressive behaviors, and prosocial altruistic tendencies in young adults. The sample consisted of 601 young adults (mean age = 19.96 years; SD = 2.27; 69.1% female) who were administered online assessments of experiences of humiliation on networks, problematic smartphone use, prosocial altruistic tendencies, and aggressiveness. Results indicated significant indirect effects of cyber victimization on aggressiveness and prosocial altruistic tendencies through problematic smartphone use. Problematic cell phone use explained the relationships between online humiliation and aggressive and prosocial altruistic behaviors. The results confirmed the positive relationship between cybervictimization and problematic cell phone use, consistent with previous research. However, the negative relationship between cybervictimization and altruistic prosocial tendencies was not corroborated. The findings emphasize the need to promote actions that foster social connectedness and interdependence among young individuals to develop their identity within the community.

## Introduction

In recent years, interest in the study of cyberbullying has significantly increased (Ferrari et al., 2019; Machmutow and Kowalski, 2020; Kuss and Griffiths, 2021; Li et al., 2021; Raza and Pasha, 2021). Cyberbullying, which was originally understood as a specific form of bullying, has gradually evolved to encompass insults, threats, humiliation, privacy violations, public disclosure of private information and refers to the deliberate harassment towards others utilizing electronic means (Watts et al., 2017). This aggression, which occurs in asymmetrical situations, can originate from an individual as well as a group (Jenaro et al., 2018), and the author is not always evident, making it challenging for the victim to defend themselves (Jenaro et al., 2007, 2018; Watts et al., 2017). This study aims to analyze, in young adults, the relationships between a specific element of cyberbullying (subjective experiences of humiliation on social networks), and problematic smartphone use along with tendencies of aggressive and altruistic behaviors.

The growing body of research associated with cyberbullying suggests that it is a concerning social phenomenon on the rise, primarily affecting young people (Qudah et al., 2019). Unlike traditional bullying, which decreases with age (Crosslin and Golman, 2014), the presence of cyberbullying tends to remain stable in secondary and tertiary education (Wensley and Campbell, 2012). It is associated with significant negative consequences for victims, such as increases in aggression (Emler and Reicher, 2005; Ak et al., 2015) and addiction (Gámez-Guadix et al., 2013; Machimbarrena et al., 2021). Although its impact on indicators of positive youth adjustment is not well understood, some studies suggest a negative relation between being a victim of cyberbullying and their prosocial behaviors. In other words, individuals who are victims of cyberbullying tend to exhibit lower levels of prosocial behavior compared to those who are not victims of cyberbullying (Kwon and Kim, 2019; Wang and Chen, 2020).

Numerous researchers have reported the existence of a tendency to exhibit hostile behavior following victimization (victim-aggressor). According to Emler et al. (1987); Emler and Reicher (1995, 2005), this paradoxical relation, where the victim develops behaviors similar to those of the aggressors, is most likely to occur when the victim does not have access to protective sources (e.g., parents, friends, teachers, authorities). Consequently, victims are driven to develop alternative forms of protection by demonstrating strength, bravery, and the ability to retaliate aggressively when attacked. Violent behaviors can become the means to defend, assuming that the aggressors cannot be victimized. Alternatively, General Strain Theory (GST) can also serve as a theoretical framework for understanding the relations between cyberbullying victimization and subsequent development of aggression (Ak et al., 2015). GST (Agnew, 1992) proposes that individuals may engage in bullying or aggressive behaviors as a response to stressful life events and the negative emotions that they produce.

The positive associations between cyber victimization and aggression has been extensively supported (Hinduja and Patchin, 2008; Ak et al., 2015; Giménez et al., 2015; ArIcak and Ozbay, 2016; Savage and Tokunaga, 2017). Recently, Martínez-Monteagudo et al. (2019), in their study differentiating between pure victims, aggressors, and victim-aggressors, demonstrated that the experience of hostile attitudes positively predicts an increased likelihood of becoming a victim-aggressor in cyberbullying. Estévez et al. (2012) analyzed the influencing factors in the transition process from victimization to involvementin aggressive behaviors (victim-aggressor) in the school context and showed that school victimization predicted subsequent violent behavior. Similarly, Schenk and Fremouw (2012) found that victims of cyberbullying exhibited higher levels of hostility. Moreover, Hoff and Mitchell (2009) reported that when victims did not know their attackers (as is the case in many social networks), anger increased significantly. Therefore, aggression is clearly linked to victimization and cyber victimization, with victims having a high likelihood of displaying later aggressive behaviors.

Contrary to the extensive research on aggression, the effect of victimization on prosocial behaviors is not as extensively studied. According to Eisenberg et al. (2015), prosocial behavior are actions through which individuals benefit others. The absence of aggression does not imply the presence of prosocial behaviors, just as the presence of aggression does not necessarily indicate an inability to display prosocial behaviors. Examples of this relation are studies that find no significant relations between prosocial behavior and aggression (e.g., Kokko et al., 2006; Gerardy et al., 2015). However, even in studies that demonstrate relations between aggressive and prosocial behaviors, the magnitude of effect sizes is moderate (e.g., Eron and Huesmann, 1984; Boxer et al., 2004; McGinley and Carlo, 2007; Carlo et al., 2014). For example, in a meta-analytic review, the overall effect size between aggressive and prosocial behaviors was moderate (r + = 0.23) and the effect varied significantly across moderator variables (such as measurement type, age, gender, SES; see Memmott-Elison et al., 2020; see also Card and Little, 2006). The mechanisms for mobilizing or inhibiting prosocial behaviors and aggressive behaviors are complex, and the overall findings suggest distinct correlates of such actions. Importantly, there is limited evidence demonstrating that both aggressors and aggressor-victims exhibit significantly lower levels of prosocial behaviors compared to uninvolved students (Marengo et al., 2018). In a study on the impact of exposure to community violence on cooperation and help tendencies, Littman et al. (2020) did not find significant relations between violence exposure and prosocial tendencies.

While there is not much research on the specific relationships between perceptions of humiliation and their consequences, studies on cyberbullying have gone hand in hand with research focused on analyzing the effects and consequences of new Information and Communication Technologies (ICTs) on the lives of young people, with one of these areas of interest being the study of problematic internet and mobile device use. The World Health Organization (WHO) uses the term “Problematic use of the Internet” (PUI) (World Health Organization, 2014) to refer to all potentially problematic behaviors related to the Internet, stating it as “a growing concern across all age groups and an emerging challenge for research” (Fineberg et al., 2018, p. 3). Excessive and harmful internet consumption has become a matter of public health on an international level (Bányai et al., 2017). In Spain, the target country of origin of the sample in the present study, addiction to new technologies has been included in the Action Plan on Addictions 2021–2024 (Ministerio de Sanidad, Servicios Sociales e Igualdad, 2021), also defining it as a public health issue.

PIU is defined as a dysfunctional use of mobile devices connected to the internet characterized by excessive use, a preference for being online rather than engaging in other activities or spending time with friends, inability to control usage time, psychological dependence, and mood regulation through the internet (Gámez-Guadix et al., 2013; Boniel-Nissim and Sasson, 2018; Zsila et al., 2018; Machimbarrena et al., 2021; Martínez-Ferrer et al., 2021). The prevalence of this phenomenon in Europe ranges from 14.3 to 54.9%, while in Spain it ranges from 2.4 to 4.95% (Hinojo-Lucena et al., 2021; Machimbarrena et al., 2021). Other studies (Qudah et al., 2019) indicate that 1 in 5 students is completely dependent on their mobile phones, and Zsila et al. (2018) found that 29.8% of the analyzed students spent more than 6 h per day on social media. Regarding gender differences in PIU, there is no consensus. Some studies show a positive trend for males as compared to females (e.g., Qudah et al., 2019), while other researcher do not find any significant gender differences (e.g., Şimşek et al., 2019). Interestingly, Martínez-Ferrer et al. (2021) suggest that girls exhibit higher problematic use of social media, leading to a higher likelihood of being cyberbullied, whereas boys tend to experience more bullying through online gaming platforms.

Compensatory Internet Use Theory (Kardefelt-Winther, 2014) suggests that the situational stressors of being bullied lead the victim to engage in compulsive use as an alternative way to cope with the negative emotions it provokes. Similarly, Problem Behavior Theory (Jessor, 1991; Machimbarrena et al., 2021) posits that young people engaged in problematic activities are more likely to engage in other risky behaviors. The environment facilitated by ICTs, where (1) anonymity generates a sense of lack of consequences for our actions, (2) immediate social reinforcement, and (3) low cost of access, make them perfect reinforcers for addictive behaviors when combined with both personal and environmental factors (Sánchez-Carbonell and Fargues, 2007; Álvarez-García et al., 2020; Llorent et al., 2021). In this regard, the literature has demonstrated a direct relation between cyberbullying and problematic smartphone use (Gámez-Guadix et al., 2013; Choi et al., 2020; Machimbarrena et al., 2021).

However, studies exploring the relationships between PIU and aggression are still limited, as evidenced by the systematic review conducted by Llorent et al. (2021), where only 2 out of a total of 44 analyzed studies examined aggression as a consequence of PIU, and both indicated that internet-addicted adolescents seem to be more prone to aggression and rule-breaking (Lim et al., 2015; Machado et al., 2018). Similarly, Nuri et al. (2021) examined the mediating role of smartphone addiction in the relationship between nomophobia and aggression levels among university students in Cyprus, demonstrating that smartphone addiction predicts hostility, physical aggression, verbal aggression, and anger. Furthermore, Naseer and Husain (2020) investigated the relationships between cellphone use and aggression among young adults in Pakistan, showing that smartphone use is associated with physical aggression, verbal aggression, expression of anger, and hostility. Likewise, it positively predicts general aggression, with this relationship being moderated by gender and marital status, with men and single users exhibiting higher levels of aggression compared to women or married cellphone users. However, findings from Sahin (2014) in university students, indicated a lack of a significant correlation between internet addiction and aggression.

If research on addiction and aggression is recent and limited, the research exploring the relations between internet addiction and its impact on positive adjustment variables such as prosocial behaviors is even scarcer, although there is suggestive recent evidence on this matter. Hao et al. (2020), interested in documenting the impacts of problematic mobile phone use on individuals’ well-being, examined its relation with altruism among university students. Their findings showed that problematic mobile phone use negatively predicted altruism both directly and indirectly through alexithymia, cognitive empathy, and affective empathy. On the other hand, López-Mora et al. (2021) demonstrated that prosocial reasoning was negatively related to the use of online communication oriented towards seeking liberation and social trust among young adults. However, the available literature does not show a significant association between prosocial behavior and the duration of mobile device use when it comes to studies involving children and adolescents (Okada et al., 2021).

Given the current evidence, as well as the rapid digitization of young people’s social interactions and the increasingly frequent experiences of cyber victimization across a broader age range, this research focuses on understanding the relationships between cyber victimization experiences and problematic smartphone use in the aggressive and prosocial tendencies of young adults. The present study, which is part of a larger project examining multiple factors, was designed to examine experiences of online humiliation and their predictive role in aggressive and prosocial behaviors among Spanish young adults, as well as the mediating role of problematic smartphone use in this phenomenon.

**Figure 1:**
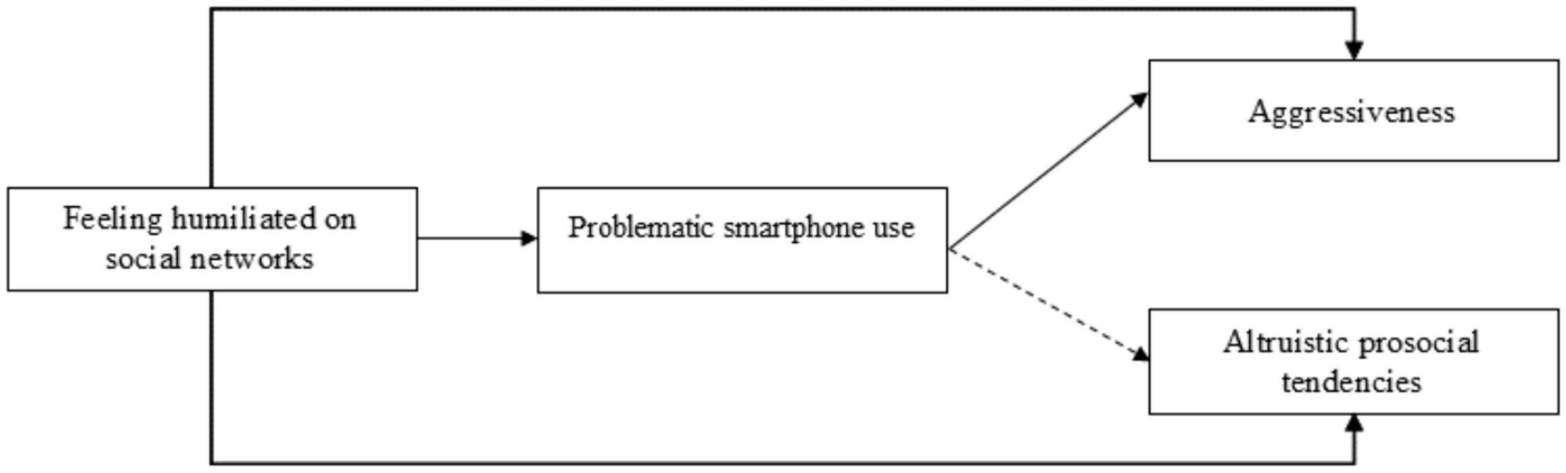
Hypothesized model.

Based on previous research, it is hypothesized that experiences of online humiliation would be positively related to problematic smartphone use (Gámez-Guadix et al., 2013; Qudah et al., 2019; Şimşek et al., 2019; Martínez-Ferrer et al., 2021), as well as aggressive behavior (Martínez-Monteagudo et al., 2019, 2020), and negatively related to prosocial behavior (Bäker and Schütz-Wilke, 2023). It is expected that, similar to humiliation, problematic smartphone use will be positively associated with indicators of aggressive behavior (Akbar et al., 2022; Zhang et al., 2022) and negatively associated with indicators of prosocial behavior (Esparza-Reig et al., 2022). Most importantly, it is expected that problematic smartphone use will mediate the association between experiences of humiliation and the aggressive and prosocial tendencies of these young adults. In other words, it is hypothesized that young people who have experienced humiliation on social media will exhibit higher levels of aggressive tendencies, lower tendencies to help others, and higher levels of problematic smartphone use (Figure 1).

## Method

### Participants

A total of 601 young adults (Mean age = 19.96 years; SD = 2.27; 69.1% female) recruited under convenience sampling at Public Universities in Spain participated in this study. The researchers had the informed consent of the participants included in the study and the approval of IRB protocols.

### Instruments

#### Sociodemographic and experiences of humiliation on social networks

Participants completed different sociodemographic data of interest (e.g., gender, age, nationality) as well as were asked to rate the frequency with which they had been humiliated/insulted on social networks using a 4-point likert scale (1 = Never; 4 = Many times).

#### Problematic smartphone use

The Problematic Mobile Phone Misuse Scale MPPUS-10; in its Spanish adaptation (López-Fernández et al., 2012) was used to assess the intensity of problematic cell phone use through the mean obtained from 10 items reflecting different problematic situations (e.g., “I find myself hooked on my cell phone longer than I would like”) that allude to elements such as tolerance, avoidance of other problems, abstinence, anxiety and negative consequences in life using a 10-point Likert-type scale anging from 1 (“not true at all”) to 10 (“extremely true”). This scale showed adequate reliability (*α* = 0.84).

#### Aggressiveness

To assess aggressiveness, participants completed the Aggression Suppression subscale of the Weinberger Adaptation Inventory (Weinberger, 1997) A total of 5 items designed to assess aggressive behaviors (example item “If someone tries to hurt me, I make sure to get even”) on a five-point scale (1 = does not describe me well to 5 = describes me very well). In this study the reliability obtained was adequate (*α* = 0.77).

#### Altruistic prosocial tendencies

Finally, 4 items were used for the altruistic subscale of a Spanish version of prosocial tendencies measure (PTM, Mestre et al., 2015). The items used (e.g., “I tend to help people who are badly hurt,” “Helping others when I am in the spotlight is when I function best”) were assessed with a 5-point scale (1 = does not describe me at all; 5 = It describes me completely) and the reliability obtained was adequate (*α* = 0.74). The measure has demonstrated acceptable reliability and validity evidence to use with adolescents and young adults from Spain (López-Mora et al., 2021).

### Procedure

Participants completed an anonymous online survey through the Qualtrix platform after being informed of the study objectives and that participation was voluntary. The data Research Topic protocols were approved by the corresponding ethics committee.

### Data analysis

Data analysis was conducted throughout various stages. First, analyses were carried out to ensure the validity of measures. Descriptive analyses (e.g., normality, mean and standard deviation) were used along with the estimation of internal consistency of the subscales using the Cronbach’s alpha coefficient and Pearson correlation analysis. The confirmatory factor analysis (CFA) procedure adjusted to maximum likelihood parameters was used to calculate the fit of the hypothesized models. The chi-square test, the comparative fit index (CFI; fi t is adequate at 0.90 or greater) and the approximation mean square error (RMSEA; fi t is adequate at 0.08 or less) were used as indicators to determine the model fit (Byrne, 2016). Finally, the chi-square increment test t (Δ *χ*^2^) and the CFI (ΔCFI; fi t is adequate at 0.01 or less) were used to estimate the model invariance by gender (Bentler and Bonett, 1980; Bentler, 1990).

## Results

### Descriptives and correlations

Table 1 shows the descriptive statistics of the scales and correlations. Feeling humiliated in social networks was positively correlated with problematic smartphone use and aggressiveness. Likewise, problematic smartphone use was positively related to aggressiveness and negatively related to altruistic prosocial tendencies and finally, it was observed that prosocial tendencies were negatively related to aggressiveness.

**Table 1:** Descriptive, correlations and mean differences between variables under study.

	M_male_ (SD)	M_female_ (SD)	t	*d*	1	2	3	4
1. Feeling humiliated on social networks	1.59 (0.86)	1.57(0.82)	0.31	0.83	*α = 0.*84			
2. Problematic smartphone use	4.32(1.84)	4.30(1.90)	0.12	1.88	0.29^**^	*α = 0.*84		
3. Altruistic prosocial tendencies	3.68(0.98)	4.11(0.85)	−5.65**	0.90	−0.04	−0.29^**^	*α = 0.*74	
4. Aggressiveness	2.23(0.82)	2.09(0.73)	2.50	0.21	0.18^**^	0.27^**^	−0.20^**^	*α = 0.*77

*p < 0.05; **p < 0.01.

### Path analysis of the main model

Path analysis were conducted made using the maximum likelihood estimation method in AMOS version 25 (Byrne, 2016). A model was executed (see Figure 1) in which the direct path between feeling humiliated on social networks, problematic smartphone use, aggressiveness and altruistic prosocial tendencies is tested. In addition, the direct path between problematic smartphone use to aggressiveness and altruistic prosocial tendencies were tested. In addition, the indirect path between feeling humiliated on social networks to aggressiveness and altruistic prosocial tendencies via problematic smartphone use were tested too.

### Main analyses

**Figure 2:**
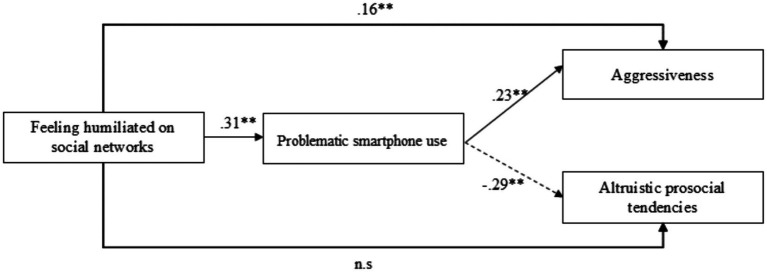
Relational model of variables.

The model fitted acceptably (*χ*^2^/DF = 7.75 *p* = 0.05; CFI = 0.95; RMSEA =0.08). Path analysis showed that feeling humiliated on social networks was positively associated with aggressiveness and problematic smartphone use se although it did not show a significant direct relation with altruistic prosocial tendencies. In addition, problematic smartphone use was positively related to aggressiveness and negatively related to altruistic prosocial tendencies (Figure 2).

### Tests of indirect effects

Bias-corrected bootstrap confidence intervals (CIs) were used to test the significance of the mediated effects (MacKinnon et al., 2002). Indirect effects were significant for relations between feeling humiliated on social networks and aggressiveness via problematic smartphone use [indirect effect = 0.08, 95% CIs = (0.04, 0.12), *p* = 0.01] feeling humiliated on social networks and altruistic prosocial tendencies via problematic smartphone use [indirect effect = −0.10, 95% CIs = (0.05, 0.10), *p* = 0.01].

## Discussion

The present research aimed to analyze the relations among cybervictimization on social networks (specifically, the subjective experience of feeling humiliated), dysfunctional use of smartphones, aggressive behaviors, and altruistic prosocial tendencies. The hypotheses posited that experiences of humiliation in an online or network context would be positively related to problematic smartphone use, as well as to aggressive behavior, and negatively related to prosocial behavior. It was also expected that problematic smartphone use would mediate the association between experiences of humiliation and the aggressive and prosocial tendencies of these young individuals.

Importantly, the findings indicated that problematic smartphone use accounted for the relations between experiences of online humiliation and both aggressive and altruistic prosocial tendencies. These findings are notable and add to our understanding of the underlying mechanisms of the association between online humiliation experiences and youth aggressive and prosocial behaviors. These findings align with the findings on aggression (e.g., Hinduja and Patchin, 2013; Zhang and Leung, 2015; Wang and Chen, 2020) and one prior study that examined effects in both aggression and prosocial behaviors (Kim and Kim, 2021). The present findings are consistent with the notion that problematic smartphone use may be a way in which young individuals express their distress and frustration after being humiliated online, which in turn may lead to increased levels of aggression and less inclination to engage in altruistic prosocial behaviors. The fact that indirect effects were found with respect to a specific form of prosocial behavior, altruistic actions that selflessly-motivated, extends prior studies that did not examine specific forms of prosocial behaviors and align with the importance of investigating specific forms of prosocial behaviors (Carlo et al., 2014). Future studies could examine whether such findings can be replicated examining other specific forms of prosocial behaviors.

The findings indicate that the hypothesis proposed regarding the positive relation between online humiliation and problematic smartphone use was supported. The relation between cybervictimization and problematic smartphone use has also been documented in previous studies (Tokunaga, 2010; Cho and Lee, 2015; Ozdemir, 2020; Gómez-Molinero et al., 2021), which have shown that individuals who experienced cyber victimization also reported excessive mobile phone use (Tokunaga, 2010; Beranuy et al., 2019; Ozdemir, 2020; Gómez-Molinero et al., 2021). Similarly, Cho and Lee (2015) found that adolescents who experienced cyber victimization were more likely to use their mobile phones compulsively. Such findings are in accord with Compensatory Internet Use Theory (Kardefelt-Winther, 2014), which suggests that persons who are victimized online are likely to also enact cell phone misuse as a means of managing such experiences.

Regarding the expected direct relations between cybervictimization and both aggression and altruistic prosocial behaviors, a positive relation between cybervictimization and aggression was confirmed but the negative relation with altruistic prosocial tendencies was not confirmed. Such findings are consistent with previous studies indicating that cybervictimization is significantly associated with an increased risk of aggressive behavior (Kowalski et al., 2008; Gámez-Guadix et al., 2013; Zych et al., 2019), delinquent behavior (Gámez-Guadix et al., 2013), and hostility (Kowalski et al., 2008). It could be that a pattern of negative cyber victimization is most directly pertinent to predicting future negative actions relatively more than undermining positive actions. Alternatively, some researchers have suggested that perhaps cyber victimization might be related to other specific forms of prosocial behavior. For example, a study by García et al. (2016) found that cybervictimization was negatively associated with direct prosocial behaviors, such as helping or sharing, but was not significantly associated with indirect prosocial behaviors, such as cooperation and empathy (similarly see Chang et al., 2019). Nonetheless, the overall pattern of findings is consistent with prior work that aggressive and prosocial behaviors are part of the same spectrum of behaviors but rather that such actions are sometimes relatively independent of each other (Carlo et al., 2014). For example, persons who frequently engage in aggressive behaviors are also capable of frequently in altruistic prosocial behaviors (e.g., gang members). This notion is also consistent with contemporary models on the multidimensional nature of both aggressive and prosocial behaviors (Carlo et al., 2014).

As expected, concerning the relations between problematic phone use and prosocial and aggressive behaviors, problematic phone use was positively related to aggression and negatively related to prosocial behaviors. These findings align with previous research that has found excessive mobile phone use to be associated with a range of negative outcomes, including direct aggression (Li, 2007; Sourander et al., 2010; Chen and Yan, 2016a) or online aggression (Hinduja and Patchin, 2013; Wang and Chen, 2020), delinquency (Li, 2007), decreased empathy (Toda et al., 2016a,b), prosocial behaviors (Wang and Chen, 2020), social skills (Zhang et al., 2022), and the quality of interpersonal relationships with friends and family (Rosen et al., 2013; Zhang et al., 2022). These findings suggest that problematic smartphone use can have negative effects on multiple areas of life.

These results should be interpreted in light of the study’s limitations. First, the measurement of variables through self-report questionnaires and the use of observable variables to establish the models have certain limitations (e.g., measurement error is not taken into account). In addition, only a single-item measure was used to assess “feeling humiliated in social networks.” Therefore, future research should employ multiple methods (e.g., multiple informants, behavioral tasks) to reduce such sources of limitation. Secondly, the sample is not representative of the general youth population. Further investigation with a community sample is necessary to better determine the generalizability of the results, also considering other variables such as time spent online or patterns of use of social media platforms and smartphones. Thirdly, the study design was cross-sectional, which does not allow for strong inferences about causality and the direction of effects and variables. The correlational nature of the study also invites testing other alternative or more complex models. It is possible, for example, that problematic mobile phone use may serve as a means of identity concealment, and when one feels that people are not directly “seeing” them, they may be more capable of engaging in aggressive behaviors. When one feels observed, the social effect reduces the tendency to act aggressively due to emotional factors and anticipated consequences. Hiding behind a mobile phone creates a sense of impunity. It is also necessary to explore whether aggressive behavior is a retaliatory response to previously inflicted harm, i.e., responding based on what the person believes the other “deserves.” It is important to note that the relation between cyber victimization and aggression can be bidirectional and complex, and studies have found that aggression can also be a predictor of cyber victimization (e.g., Ong et al., 2014; Gini et al., 2018; Zych et al., 2019). This, future research is needed that directly examines possible bidirectional relations between cyber victimization and aggression.

Despite these limitations of the study, the present findings contribute to our understanding of the consequences of cyber victimization, problematic mobile phone use, and the resulting prosocial and aggressive behaviors. Given the relatively limited research examining these relationships, the findings provide evidence that cyber victimization has consequences beyond the experience of harassment itself and highlight the importance of promoting actions that foster social connection and interdependence among young individuals as a means to develop their personal identity within the community. This can help convey the idea that no one exists as an isolated and independent individual, but rather, we are all interconnected (both in real life and beyond the online realm), and that one’s behavior at any given moment has an exponentially expanding effect in all directions. That behavior has an impact both within and outside the online environment. Although further research is needed, program developers and policymakers may consider supporting interventions that encourage responsible use of online platforms.

Specifically, the present findings have important practical implications for professionals, educators, parents, and caregivers working with youth and adolescents. The evidence presented suggests that cyber victimization on social networks is associated with increased aggression and problematic mobile phone use. Since aggression and problematic mobile phone use are detrimental to the mental health and emotional well-being of young individuals, professionals and educators should consider developing prevention and treatment programs targeting these areas. The programs could include activities designed to improve conflict resolution and social skills, with the aim of reducing the risk of online victimization and problematic mobile phone use. Ultimately, the results of this study highlight the importance of taking steps to reduce cyber victimization and promote the mental health and emotional well-being of young individuals. Effective prevention and treatment of cyber victimization through psychoeducation and training in skills to use online content responsibly can help reduce long-term negative effects.

## Data availability statement

The original contributions presented in the study are included in the article/supplementary material, further inquiries can be directed to the corresponding author.

## Ethics statement

The studies involving humans were approved by the Universidad Europea de Valencia. The studies were conducted in accordance with the local legislation and institutional requirements. The participants provided their written informed consent to participate in this study.

## Author contributions

CL-M: Conceptualization, Data curation, Formal analysis, Funding acquisition, Investigation, Methodology, Project administration, Resources, Software, Supervision, Validation, Visualization, Writing – original draft, Writing – review & editing. GC: Conceptualization, Investigation, Project administration, Resources, Supervision, Writing – review & editing. IHL: Conceptualization, Investigation, Supervision, Visualization, Writing – review & editing. FG-B: Investigation, Methodology, Software, Supervision, Writing – review & editing. EOG: Supervision, Visualization, Writing – review & editing.

## References

[ref1] Agnew R. (1992). Foundation for a general strain theory of crime and delinquency. Criminology 30, 47–88. doi: 10.1111/j.1745-9125.1992.tb01093.x

[ref2] Ak Ş. Özdemir Y. Kuzucu Y. (2015). Cybervictimization and cyberbullying: the mediating role of anger, don’t anger me! Comput. Hum. Behav. 49, 437–443. doi: 10.1016/j.chb.2015.03.030

[ref3] Akbar F. Ahsan S. Andleeb S. N. Khan S. (2022). Mediating role of family relations between internet addiction and aggression among university students. Pak. J. Psychol. Res. 37, 417–434. doi: 10.33824/PJPR.2022.37.3.25

[ref4] Álvarez-García D. Barreiro-Collazo A. Núñez J. C. González-Castro P. Rodríguez C. (2020). Cyberbullying victimization, self-esteem and suicidal ideation in adolescence: does emotional intelligence play a buffering role? Front. Psychol. 11:3033. doi: 10.3389/fpsyg.2020.573307 PMC5874900 29623058

[ref5] ArIcak O. T. Ozbay A. (2016). Investigation of the relationship between cyberbullying, cybervictimization, alexithymia and anger expression styles among adolescents. Comput. Hum. Behav. 55, 278–285. doi: 10.1016/J.CHB.2015.09.015

[ref6] Bäker N. Schütz-Wilke J. (2023). Behavioral changes during the first year of the COVID-19 pandemic: A longitudinal comparison of bullying, cyberbullying, externalizing behavior problems and prosocial behavior in adolescents. COVID 3, 289–300. doi: 10.3390/covid3020022

[ref7] Bányai F. Zsila Á. Király O. Maraz A. Elekes Z. Griffiths M. D.. (2017). Problematic social media use: results from a large-scale nationally representative adolescent sample. PLoS One 12:e0169839. doi: 10.1371/journal.pone.0169839, PMID: 28068404 PMC5222338

[ref8] Bentler P. M. (1990). Comparative fi t indexes in structural models. Psychol. Bull. 107, 238–246. doi: 10.1037/0033-2909.107.2.238 2320703

[ref9] Bentler P. Bonett D. (1980). Signifi cance tests and goodness of fit in the analysis of covariance structures. Psychol. Bull. 88, 588–606. doi: 10.1037/0033-2909.88.3.588

[ref10] Beranuy M. Carbonell X. Griffiths M. D. (2019). A qualitative analysis of online gaming addicts in treatment. Int. J. Ment. Heal. Addict. 17, 1007–1021. doi: 10.1007/s11469-019-00088-7

[ref11] Boniel-Nissim M. Sasson H. (2018). Bullying victimization and poor relationships with parents as risk factors of problematic internet use in adolescence. Comput. Hum. Behav. 88, 176–183. doi: 10.1016/j.chb.2018.05.041

[ref12] Boxer P. Tisak M. S. Goldstein S. E. (2004). Is it bad to be good? An exploration of aggressive and prosocial behavior subtypes in adolescence. J. Youth Adolesc. 33, 91–100. doi: 10.1023/B:JOYO.0000013421.02015.ef

[ref13] Byrne B. M. (2016). Structural equation modeling with AMOS: Basic concepts, applications, and programming. 3rd Edn. New York: Routledge.

[ref14] Card N. A. Little T. D. (2006). Proactive and reactive aggression in childhood and adolescence: A meta-analysis of differential relations with psychosocial adjustment. Int. J. Behav. Dev. 30, 466–480. doi: 10.1177/0165025406071904

[ref15] Carlo G. Mestre M. V. McGinley M. M. Tur-Porcar A. Samper P. Opal D. (2014). The protective role of prosocial behaviors on antisocial behaviors: the mediating effects of deviant peer affiliation. J. Adolesc. 37, 359–366. doi: 10.1016/j.adolescence.2014.02.009, PMID: 24793382

[ref16] Chang F.-C. Yan J.-R. Huang Y.-M. (2019). The mediating role of psychological distress in the relationship between cybervictimization and prosocial behavior among adolescents. J. Interpers. Violence 36:NP3036–NP3056. doi: 10.1177/0886260517729845

[ref17] Chen L. Yan Z. (2016a). Online and offline social rejection: social network sites, social exclusion, and aggression. J. Educ. Comput. Res. 54, 1056–1072. doi: 10.1177/0735633116650463

[ref18] Cho S.-H. Lee K. M. (2015). How do adolescents cope with cyber victimization? The role of emotion regulation, coping efficacy, and hope on the relationship between cyber victimization and psychological adjustment. Comput. Hum. Behav. 49, 62–73. doi: 10.1016/j.chb.2015.01.071

[ref19] Choi J. Lee W. Kim H. (2020). The impact of cyberbullying victimization on mobile phone addiction among adolescents: the moderating effect of gender. Child Youth Serv. Rev. 111:104860. doi: 10.1016/j.childyouth.2020.104860

[ref20] Crosslin K. Golman M. (2014). “Maybe you don’t want to face it” - college students’ perspectives on cyberbullying. Comput. Hum. Behav. 41, 14–20. doi: 10.1016/j.chb.2014.09.007

[ref21] Eisenberg N. Spinrad T. L. Knafo-Noam A. (2015). “Prosocial development, Handbook of child psychology and developmental science” in Socioemotional processes. eds. Lamb M. E. Lerner R. M., vol. 3. 7th ed (New Jersey: Wiley), 610–656.

[ref22] Emler N. Reicher S. (1995). Adolescence and delinquency: the collective management of reputation. Cambridge: Blackwell Publishing.

[ref23] Emler N. Reicher S. (2005). Delinquency: cause or consequence of social exclusion? En Abrams D. Marques J. Hogg Y M. (Eds.): The social psychology of inclusion and exclusion (pp. 211–241). Philadelphia: Psychology Press.

[ref24] Emler N. Reicher S. Ross A. (1987). The social context of delinquent conduct. J. Child Psychol. Psychiatry 28, 99–109. doi: 10.1111/j.1469-7610.1987.tb00655.x 3558543

[ref25] Eron L. D. Huesmann L. R. (1984). The relation of prosocial behavior to the development of aggression and psychopathology. Aggress. Behav. 10, 201–211. doi: 10.1002/1098-2337(1984)10:3<201::AID-AB2480100304>3.0.CO;2-S

[ref27] Esparza-Reig J. Martí-Vilar M. Merino-Soto C. García-Casique A. (2022). Relationship between prosocial Behaviours and addiction problems: A systematic review. Healthcare 10:74. doi: 10.3390/healthcare10010074 PMC8774983 35052238

[ref28] Estévez E. Inglés C. J. Emler N. P. Martínez-Monteagudo M. C. Torregrosa M. S. (2012). Análisis de la relación entre la victimización y la violencia escolar: El rol de la reputación antisocial. Psychosoc. Interv. 21, 53–65. doi: 10.5093/in2012v21n1a3

[ref29] Ferrari G. Lazzari D. Di Blasio P. (2019). Cyberbullying among university students: A systematic review. Int. J. Environ. Res. Public Health 16:3655. doi: 10.3390/ijerph16193655 31569445 PMC6801478

[ref30] Fineberg N. A. Demetrovics Z. Stein D. J. Ioannidis K. Potenza M. N. Grünblatt E.. (2018). Manifesto for a European research network into problematic usage of the internet. Eur. Neuropsychopharmacol. 28, 1232–1246. doi: 10.1016/j.euroneuro.2018.08.004 30509450 PMC6276981

[ref31] Gámez-Guadix M. Orue I. Smith P. K. Calvete E. (2013). Longitudinal and reciprocal relations of cyberbullying with depression, substance use, and problematic internet use among adolescents. J. Adolesc. Health 53, 446–452. doi: 10.1016/j.jadohealth.2013.03.030, PMID: 23721758

[ref33] García T. Romero E. Beranuy M. Fernández-Montalvo J. (2016). How does social support mitigate the impact of severe forms of cyberbullying? The mediating role of emotion-focused coping strategies. Int. J. Clin. Health Psychol. 16, 151–159.

[ref34] Gerardy H. Mounts N. S. Luckner A. E. Valentiner D. P. (2015). Mothers' management of adolescent peer relationships: associations with aggressive, prosocial, and playful behavior. J. Genet. Psychol. 176, 299–314. doi: 10.1080/00221325.2015.1066746, PMID: 26244710

[ref35] Giménez A. M. Maquilón J. J. Arnaiz P. (2015). Usos problemáticos y agresivos de las TIC por parte de adolescentes implicados en cyberbullying. Revista de Investigación Educativa 33, 335–351. doi: 10.6018/RIE.33.2.199841

[ref36] Gini G. Card N. A. Pozzoli T. (2018). A meta-analysis of the differential relations of traditional and cyber-victimization with internalizing problems. Aggress. Behav. 44, 185–198. doi: 10.1002/ab.21742, PMID: 29160943

[ref37] Gómez-Molinero R. Casas-Jiménez M. Ortega-Ruiz R. (2021). Cybervictimization and problematic use of Mobile phones in adolescence: the mediating role of anxiety and depression. Int. J. Environ. Res. Public Health 18:3978.33918887

[ref38] Hao Z. Jin L. Lyu R. Rabia Akram H. (2020). Problematic mobile phone use and altruism in Chinese undergraduate students: the mediation effects of alexithymia and empathy. Child Youth Serv. Rev. 118:105402. doi: 10.1016/J.CHILDYOUTH.2020.105402

[ref39] Hinduja S. Patchin J. W. (2008). Offline consequences of online victimization. J. School. Viol. 6, 89–112. doi: 10.1300/J202v06n03_06

[ref40] Hinduja S. Patchin J. W. (2013). Social influences on cyberbullying behaviors among middle and high school students. J. Youth Adolesc. 42, 711–722. doi: 10.1007/s10964-012-9902-4, PMID: 23296318

[ref41] Hinojo-Lucena F. J. Aznar-Díaz I. Trujillo-Torres J. M. Romero-Rodríguez J. M. (2021). Problematic internet use and psychological or physical variables in university students. Revista Electronica de Investigacion Educativa 23, 1–17. doi: 10.24320/redie.2021.23.e13.3167

[ref42] Hoff D. L. Mitchell S. N. (2009). Cyberbullying: causes, effects, and remedies. J. Educ. Adm. 47, 652–665. doi: 10.1108/09578230910981107

[ref43] Jenaro C. Flores N. Gómez-Vela M. González-Gil F. Caballo C. (2007). Problematic internet and cell-phone use: psychological, behavioral, and health correlates. Addict. Res. Theory 15, 309–320. doi: 10.1080/16066350701350247

[ref44] Jenaro C. Flores N. Orgaz B. Cruz M. Pérez-Rodríguez M. Á. Vega V. (2018). Problematic internet and cell-phone use: psychological, behavioral, and health correlates. Addict. Res. Theory 26, 387–397. doi: 10.1080/16066359.2018.1429282

[ref45] Jessor R. (1991). Risk behavior in adolescence: A psychosocial Framewrok for understanding and action. J. Adolesc. Health 12, 597–605. doi: 10.1016/1054-139X(91)90007-K, PMID: 1799569

[ref46] Kardefelt-Winther D. (2014). A conceptual and methodological critique of internet addiction research: towards a model of compensatory internet use. Comput. Hum. Behav. 31, 351–354. doi: 10.1016/j.chb.2013.10.059

[ref47] Kim M. Kim J. H. (2021). Mobile phone use, social support, and subjective well-being among young adults: A longitudinal mediation model. Comput. Hum. Behav. 122:106837. doi: 10.1016/j.chb.2021.106837

[ref49] Kokko K. Tremblay R. E. Lacourse E. Nagin D. S. Vitaro F. (2006). Trajectories of prosocial behavior and physical aggression in middle childhood: links to adolescent school dropout and physical violence. J. Res. Adolesc. 16, 403–428. doi: 10.1111/j.1532-7795.2006.00500.x

[ref50] Kowalski R. M. Limber S. P. Agatston P. W. (2008). Cyber bullying: Bullying in the digital age. New Jersey: John Wiley & Sons.

[ref51] Kuss D. J. Griffiths M. D. (2021). A systematic review of cyberbullying research: definition, prevalence, risk factors, and interventions. J. Adolesc. Health 68, S4–S5. doi: 10.1016/j.jadohealth.2020.12.015

[ref52] Kwon J. Kim J. (2019). The relationship between cyberbullying victimization and prosocial behavior: the moderating effect of emotional intelligence. J. Sch. Psychol. 70, 1–14.

[ref53] Li Q. (2007). New bottle but old wine: A research of cyberbullying in schools. Comput. Hum. Behav. 23, 1777–1791. doi: 10.1016/j.chb.2005.10.005

[ref54] Li Q. Li X. Li D. Du Y. (2021). A systematic review of cyberbullying research: prevalence, risk factors, and interventions. J. Adolesc. Health 68, 53–56. doi: 10.1016/j.jadohealth.2020.10.008 33183926

[ref9001] Lim J. A. Gwak A. R. Park S. M. Kwon J. G. Lee J. Y. Jung H. Y.. (2015). Are adolescents with internet addiction prone to aggressive behavior? The mediating effect of clinical comorbidities on the predictability of aggression in adolescents with internet addiction. Cyberpsychol Behav Soc Netw. 18, 260–267. doi: 10.1089/cyber.2014.0568 25902276 PMC4432783

[ref55] Littman R. Estrada S. Stagnaro M. N. Dunham Y. Rand D. Baskin-Sommers A. (2020). Violencia comunitaria y prosocialidad: Experimentar y cometer violencia predice el castigo que impone las normas, pero no la cooperación. Ciencias de la personalidad y psicología social 11, 276–283.

[ref56] Llorent V. J. Diaz-Chaves A. Zych I. Twardowska-Staszek E. Marín-López I. (2021). Bullying and cyberbullying in Spain and Poland, and their relation to social, emotional and Moral competencies. Sch. Ment. Heal. 13, 535–547. doi: 10.1007/s12310-021-09473-3

[ref58] López-Fernández O. Honrubia-Serrano M. L. Freixa-Blanxart M. (2012). Adaptación española del" mobile phone problem use scale" para población adolescente. Adicciones 24, 123–130. doi: 10.20882/adicciones.104, PMID: 22648315

[ref59] López-Mora C. Carlo G. Roos J. Maiya S. González-Hernández J. (2021). Perceived attachment and problematic smartphone use in young people: mediating effects of self-regulation and prosociality. Psicothema 33, 564–570. doi: 10.7334/PSICOTHEMA2021.60, PMID: 34668470

[ref9002] Machado M. D. R. Bruck I. Antoniuk S. A. Cat M. N. L. Soares M. C. Silva A. F. D. (2018). Internet addiction and its correlation with behavioral problems and functional impairments–A cross-sectional study. J. Bras. Psiquiatr. 67, 34–38. doi: 10.1590/0047-2085000000181

[ref60] Machimbarrena J. M. González-Cabrera J. Montiel I. Ortega-Barón J. (2021). An exploratory analysis of different problematic internet use profiles in Cybervictims, cyberbullies, and cyberbully victims. Cyberpsychol. Behav. Soc. Netw. 24, 664–672. doi: 10.1089/cyber.2020.0545, PMID: 33606563

[ref61] Machmutow K. Kowalski R. M. (2020). A systematic review of cyberbullying among adolescents and young adults: prevalence, risk factors, and interventions. Comput. Hum. Behav. 100, 93–104. doi: 10.1016/j.chb.2019.06.017

[ref62] MacKinnon D. P. Lockwood C. M. Hoffman J. M. West S. G. Sheets V. (2002). A comparison of methods to test mediation and other intervening variable effects. Psychol. Methods 7, 83–104. doi: 10.1037/1082-989x.7.1.83, PMID: 11928892 PMC2819363

[ref63] Marengo D. Jungert T. Iotti N. O. Settanni M. Thornberg R. Longobardi C. (2018). Conflictual student–teacher relationship, emotional and behavioral problems, prosocial behavior, and their associations with bullies, victims, and bullies/victims. Educ. Psychol. 38, 1201–1217. doi: 10.1080/01443410.2018.1481199

[ref64] Martínez-Ferrer B. León-Moreno C. Suárez-Relinque C. del Moral-Arroyo G. Musitu-Ochoa G. (2021). Cybervictimization, offline victimization, and cyberbullying: the mediating role of the problematic use of social networking sites in boys and girls. Psychosoc. Interv. 30, 155–162. doi: 10.5093/PI2021A5

[ref65] Martínez-Monteagudo M. C. Delgado B. García-Fernández J. M. Rubio E. (2019). Cyberbullying, aggressiveness, and emotional intelligence in adolescence. Int. J. Environ. Res. Public Health 16:5079. doi: 10.3390/ijerph16245079 31842418 PMC6950617

[ref9004] Martínez-Monteagudo M. C. Delgado B. Inglés C. J. Escortell R. (2020). Cyberbullying and social anxiety: a latent class analysis among Spanish adolescents. Int. J. Environ. Res. Public Health. 17:406. doi: 10.3390/ijerph17020406 31936243 PMC7013764

[ref67] McGinley M. Carlo G. (2007). Two sides of the same coin? The relations between prosocial and physically aggressive behaviors. J. Youth Adolesc. 36, 337–349. doi: 10.1007/s10964-006-9095-9, PMID: 27519032

[ref68] Memmott-Elison M. K. Holmgren H. G. Padilla-Walker L. M. Hawkins A. J. (2020). Associations between prosocial behavior, externalizing behaviors, and internalizing symptoms during adolescence: A meta-analysis. J. Adolesc. 80, 98–114. doi: 10.1016/j.adolescence.2020.01.012, PMID: 32087386

[ref9005] Mestre M. V. Carlo G. Samper P. Tur-Porcar A. M. Mestre A. L. (2015). Psychometric evidence of a multidimensional measure of prosocial behaviors for spanish adolescents. J. Genet. Psychol. 176, 260–271. doi: 10.1080/00221325.2015.1052726 26132507

[ref69] Ministerio de Sanidad, Servicios Sociales e Igualdad. (2021). Plan de Acción sobre Adicciones 2021–2024. Available at: https://pnsd.sanidad.gob.es/pnsd/planAccion/docs/PlanASA_2021-24_aprobado.pdf

[ref70] Naseer A. Husain W. (2020). The relationship of cell phone use and aggression among young adults with moderating roles of gender and marital status. Insights Depress Anxiety. 4, 055–058. doi: 10.29328/journal.ida.1001020

[ref9006] Nuri C. Direktör C. Arnavut A. (2021). The mediation effects of smartphone addiction on relationship between aggression and nomophobia. World J. Educ. Technol.: Curr. Issues. 13. doi: 10.18844/wjet.v13i1.5403106-114

[ref71] Okada S. Doi S. Isumi A. Fujiwara T. (2021). The association between mobile devices use and behavior problems among fourth grade children in Japan. Psychiatry Clin. Neurosci. 75, 286–293. doi: 10.1111/PCN.13283 34176185

[ref72] Ong Y. H. Ang R. P. Ho J. C. M. Lim J. C. Y. Goh D. H. Lee C. S.. (2014). Narcissism, extraversion and adolescents' self-presentation on Facebook. Personal. Individ. Differ. 69, 176–181. doi: 10.1016/j.paid.2014.05.032

[ref73] Ozdemir M. (2020). The association between cyber victimization and problematic smartphone use among adolescents: the mediating role of fear of missing out and the moderating role of gender. Curr. Psychol. 39, 1625–1633. doi: 10.1007/s12144-018-9912-8

[ref74] Qudah M. F. Albursan I. S. Bakhiet S. F. A. Hassan E. M. A. H. Alfnan A. A. Aljomaa S. S.. (2019). Smartphone addiction and its relationship with cyberbullying among university students. Int. J. Ment. Heal. Addict. 17, 628–643. doi: 10.1007/s11469-018-0013-7

[ref75] Raza S. Pasha O. (2021). A systematic review of cyberbullying among college students: prevalence, risk factors, and interventions. J. Educ. Technol. Dev. Exchange 14, 1–18. doi: 10.33423/jetde.v14i1.3460

[ref77] Rosen L. D. Whaling K. Rab S. Carrier L. M. Cheever N. A. (2013). Is Facebook creating “iDisorders”? The link between clinical symptoms of psychiatric disorders and technology use, attitudes and anxiety. Comput. Hum. Behav. 29, 1243–1254. doi: 10.1016/j.chb.2012.11.012

[ref9007] Sahin M. (2014). The internet addiction and aggression among university students. Dusunen Adam - J. Psychiatry Neurol. Sci. 27:43. doi: 10.5350/DAJPN2014270106

[ref78] Sánchez-Carbonell X. Fargues M. B. (2007). “La adicción a Internet como sobreadaptación social” in Globalización y salud mental. Ed. A. Talarn (Barcelona: Herder), 341–368.

[ref79] Savage M. W. Tokunaga R. S. (2017). Moving toward a theory: testing an integrated model of cyberbullying perpetration, aggression, social skills, and internet self-efficacy. Comput. Hum. Behav. 71, 353–361. doi: 10.1016/j.chb.2017.02.016

[ref80] Schenk A. M. Fremouw W. J. (2012). Prevalence, psychological impact, and coping of cyberbully victims among college students. J. Sch. Violence 11, 21–37. doi: 10.1080/15388220.2011.630310

[ref81] Şimşek N. Şahin D. Evli M. (2019). Internet addiction, cyberbullying, and victimization relationship in adolescents: A sample from Turkey. J. Addict. Nurs. 30, 201–210. doi: 10.1097/JAN.0000000000000296, PMID: 31478968

[ref82] Sourander A. Klomek A. B. Ikonen M. Lindroos J. Luntamo T. Koskelainen M.. (2010). Psychosocial risk factors associated with cyberbullying among adolescents: a population-based study. Arch. Gen. Psychiatry 67, 720–728. doi: 10.1001/archgenpsychiatry.2010.79 20603453

[ref83] Toda M. Ezoe S. Mure K. Nagasawa S. Shimamura K. Koda M.. (2016a). Negative impact of social exclusion on the empathy for pain in basic level of the perceptual-motor process. Front. Psychol. 7:677. doi: 10.3389/fpsyg.2016.00677 27242591 PMC4860507

[ref84] Toda M. Monden K. Kubo K. Morimoto K. (2016b). Mobile phone dependence and health-related lifestyle of university students. Soc. Behav. Pers. 44, 617–626. doi: 10.2224/sbp.2016.44.4.617

[ref85] Tokunaga R. S. (2010). Following you home from school: A critical review and synthesis of research on cyberbullying victimization. Comput. Hum. Behav. 26, 277–287. doi: 10.1016/j.chb.2009.11.014

[ref86] Wang J. Chen S. (2020). The association between cyberbullying victimization and prosocial behavior: A meta-analytic review. J. Sch. Psychol. 74, 1–14.

[ref88] Watts L. K. Wagner J. Velasquez B. Behrens P. I. (2017). Cyberbullying in higher education: A literature review. Comput. Hum. Behav. 69, 268–274. doi: 10.1016/j.chb.2016.12.038

[ref89] Weinberger D. A. (1997). Distress and self-restraint as measures of adjustment across the life span: confirmatory factor analyses in clinical and nonclinical samples. Psychol. Assess. 9, 132–135. doi: 10.1037/1040-3590.9.2.132

[ref90] Wensley K. Campbell M. (2012). Heterosexual and non-heterosexual young university students’ involvement in traditional and cyber forms of bullying. Cyberpsychol Behav Soc Netw. 15, 649–654. doi: 10.1089/cyber.2012.0132 23078337

[ref91] World Health Organization. (2014). Problematic use of the internet. Avaialable at: https://www.who.int/publications/i/item/9789241509367

[ref92] Zhang Y. Hou Z. Wu S. Li X. Hao M. Wu X. (2022). The relationship between internet addiction and aggressive behavior among adolescents during the COVID-19 pandemic: anxiety as a mediator. Acta Psychol. 227:103612. doi: 10.1016/j.actpsy.2022.103612, PMID: 35598380 PMC9091340

[ref93] Zhang H. Leung L. (2015). Personality factors and social media use in China. Personal. Individ. Differ. 76, 161–165. doi: 10.1016/j.paid.2014.12.017

[ref94] Zsila Á. Orosz G. Király O. Urbán R. Ujhelyi A. Jármi É.. (2018). Psychoactive substance use and problematic internet use as predictors of bullying and cyberbullying victimization. Int. J. Ment. Heal. Addict. 16, 466–479. doi: 10.1007/s11469-017-9809-0, PMID: 29670501 PMC5897465

[ref95] Zych I. Baldry A. C. Farrington D. P. (2019). Bullies, victims, and bully–victims in late adolescence: symptomatology, social factors, and substance use. J. Adolesc. 72, 124–131. doi: 10.1016/j.adolescence.2019.03.003 30884429

